# Strain-tunable electronic transport in MXenes for sensing and stable electronics

**DOI:** 10.1038/s41598-026-40587-3

**Published:** 2026-02-16

**Authors:** Omid Soltani, Mohammad Reza Jafari

**Affiliations:** Department of Condensed Matter Physics, Faculty of Physics, Alzahra University, Tehran, Iran

**Keywords:** MXene, Landauer–Büttiker formalism, Tight-binding, Electronic transport, Strain engineering, Low D materials, Engineering, Materials science, Physics

## Abstract

In this paper, we present strain-tunable electronic transport in two functionalized MXenes, Ti₃C₂O₂ and Sc₃C₂F₂, using a parametric tight-binding Hamiltonian within the Landauer–Büttiker formalism. The electrode self-energies were obtained via the Sancho-Rubio recursive method, which ensures stable numerical behavior of semi-infinite electrodes. Uniaxial tensile/compressive strains were applied in the in-plane and out-of-plane directions, and their effects on the density of states (DOS), transmission, and current-voltage (I-V) response were analyzed. The results indicate that the effects of strain on the Ti_3_C_2_O_2_ structure cause a decrease in the band gap, an increase in conductivity, and a sensitivity of current to strain, making it a suitable candidate for pressure sensors. In contrast, Sc_3_C_2_F_2_ is resistant to strain, making it a good candidate for reliable flexible electronics.

## Introduction

 MXenes, a rapidly growing family of two-dimensional transition-metal carbides and nitrides, have attracted tremendous attention due to their remarkable combination of metallic conductivity, structural flexibility, and chemical tunability^1–3^. Since their first discovery in 2011, MXenes have been explored for.

applications ranging from energy storage and catalysis to nanoelectronics and sensing devices^4–8^. Among them, functionalized MXenes such as Ti₃C₂O₂ and Sc₃C₂F₂ exhibit highly diverse electronic properties depending on the surface terminations, offering a fertile ground for tailoring charge transport characteristics at the nanoscale^9,10^.

The electronic properties of MXenes are so interesting and important that a book has been written about them^11^. Many MXenes have high metallic conductivity, making them ideal for making electrodes and transmission channels in nanoelectronic devices. On the other hand, previous findings have shown that shrinking the dimensions of MXene structures often provides a suitable energy gap for their use in electronic devices^12–16^.

One compelling and experimentally feasible approach is strain engineering, which has been extensively applied in graphene, transition-metal dichalcogenides, and black phosphorus to induce bandgap modulation, control carrier mobility, and even trigger metal–insulator transitions. For MXenes, their flexible layered nature makes them particularly suitable for strain-induced tuning of properties; however, studies of strain effects on quantum transport, such as density of states, transitions at non-zero temperatures, are still scarce^17–19^. For example, by adjusting the thickness of the MXenes layer and applying vertical strain to its layer, they have concluded that the electronic properties of multilayer Ti_2_CO_2_ can be effectively manipulated by strain, and the multilayer configurations can be used in strain sensors. Applying strain can also affect the tunable optical absorption properties of MXenes^20,21^.

In the context of nanoelectronics device design, it is essential to understand how uniaxial strain affects the current-voltage (I-V) characteristics, density of states (DOS), and its impact on phenomena such as negative differential resistance (NDR) and its application in nanoelectronics. Strain not only changes the interatomic distances and hopping parameters in tight-coupling Hamiltonians, but also can make the states available for electrical conduction under finite bias available. As a result, it offers a route to control the performance of nanoelectronics devices. Such tunability is highly desirable for applications in nanoswitches, strain sensors, logic devices, and flexible electronics^22–28^.

In this paper, we first examine the effects of strain in horizontal and vertical directions on the total density of states. Then, to better understand the results, the projected density of states (PDOS) and transmission spectra were considered. Next, we evaluate the effects of strain on the current-voltage diagram of the two mentioned structures and show that strain can play an important role in the transport structure of the MXenes device.

## Computational details

**Fig. 1:**
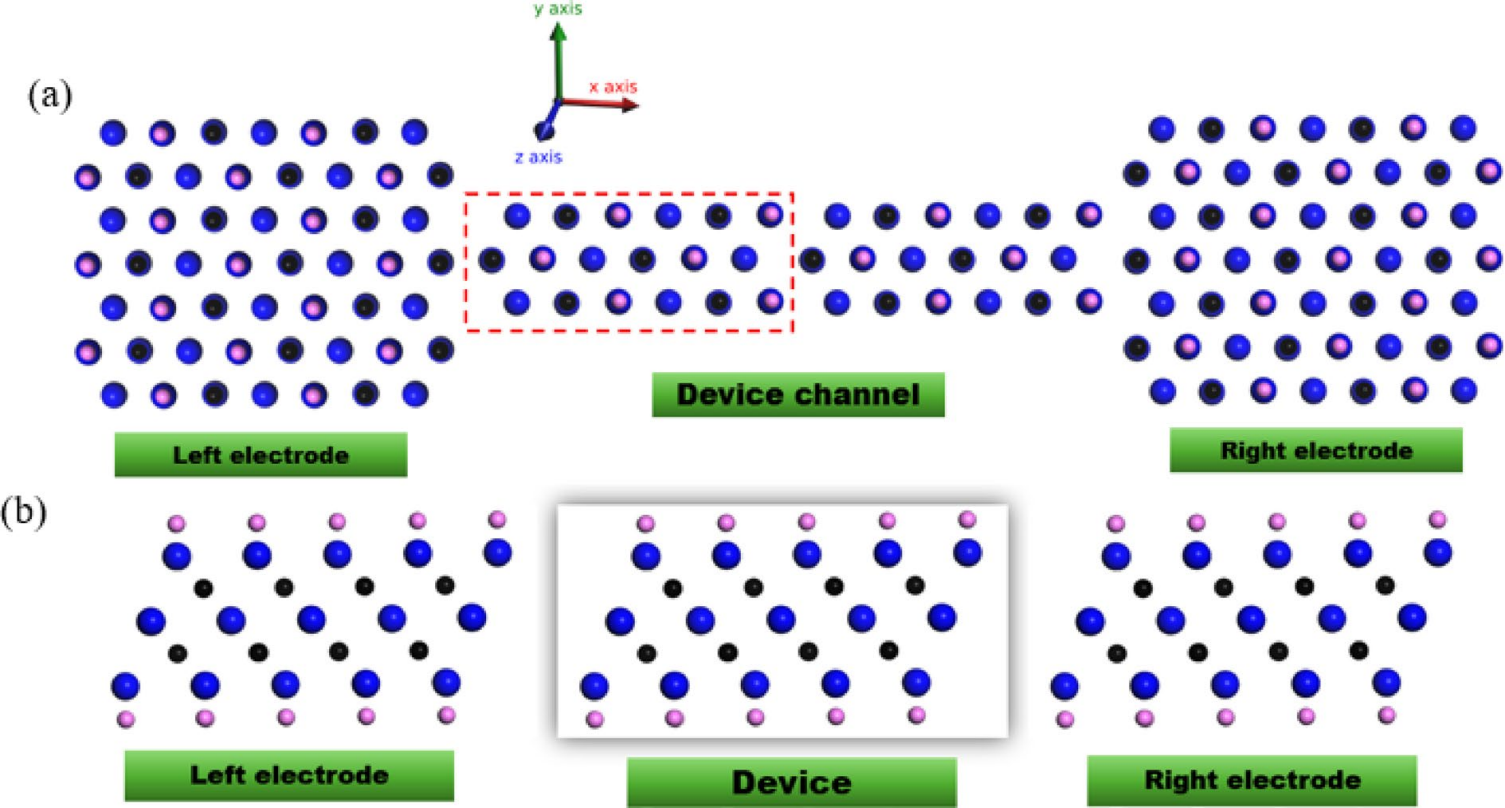
(**a**) top view and (**b**) side view of M_3_C_2_T_2_ MXene device schematic. The metal atom is shown in blue, the carbon atom in black, and the surface terminator in pink. The unit cell is shown in the red dashed box.

The simulated device consists of a central scattering region connected to two semi‑infinite electrodes. The central region (highlighted by a dashed box in Fig. 1a) contains 42 atoms (18 M, 12 C, 12 T). Fig. 1b shows the side view of the device structure under investigation. The central scattering region is highlighted by a red dashed box.This atomic unit forms a periodic supercell that is repeated to construct a quasi‑1D nanoribbon channel, a standard model for investigating ballistic electron transport in 2D materials. The electrodes are made of the same MXene material, extending infinitely to model ideal metallic contacts. Modeling the device with this fundamental repeating unit allows us to exploit its translational symmetry, significantly reducing computational complexity while preserving the essential chemistry needed to capture qualitative strain-induced trends. This computational setup is designed to isolate and probe the intrinsic electronic response of the MXene to applied strain, making it directly relevant to understanding its behavior in flexible electronics and strain‑sensing applications. The tight-binding parameters used in this work are listed in Table 1. They include first- and second-neighbor hopping integrals (t_ij_) and on-site energies (ε_i_) for Ti₃C₂O₂ and Sc₃C₂F₂. These parameters were obtained from density functional theory (DFT) calculations and have been successfully applied in our previous studies of MXene electronic structure^12,14^.

**Table 1:** The Slater-Koster parameters in units of eV for $S{c_3}{C_2}{F_2}$and $T{i_3}{C_2}{O_2}$ structures^12–14^.

SK parameter	$T{i_3}{C_2}{O_2}$	$S{c_3}{C_2}{F_2}$
${E_z}$	-1.100	-0.963
${E_x}={E_y}$	-1.534	-1.062
${E_{{x^2} - {y^2}}}={E_{xy}}\,(center\,M\,atom)$	2.817	5.344
${E_{{z^2}}}(center\,M\,atom)$	2.091	5.479
${E_{yz}}={E_{zx}}\,(center\,M\,atom)$	4.392	6.513
${E_{{x^2} - {y^2}}}={E_{xy}}\,$	2.319	4.344
${E_{{z^2}}}$	2.195	3.979
${E_{yz}}={E_{zx}}\,$	4.081	5.213
$pp\sigma$	0.282	0.390
$pp\pi$	0.178	-0.047
$dd\sigma (1NN)$	-0.719	1.279
$dd\pi (1NN)$	0.553	-0.239
$dd\delta (1NN)$	-0.226	-0.164
$dd\sigma (2NN)$	-0.439	0.472
$dd\pi (2NN)$	-0.111	0.419
$dd\delta (2NN)$	0.050	-0.027
$dd\sigma (center\,M\,atom)$	-0.563	-0.389
$dd\pi (center\,M\,atom)$	-0.129	0.073
$dd\delta (center\,M\,atom)$	0.050	0.102
$pd\sigma (1NN)$	0.430	-0.117
$pd\pi (1NN)$	0.268	-0.111
$pd\sigma (2NN)$	0.591	-0.131
$pd\pi (2NN)$	0.716	-0.356

The tight-binding parameters employed here are obtained from DFT calculations and used within the coherent Landauer–Büttiker framework. While this approach reliably captures qualitative trends induced by strain in the low-bias regime (V ≤ 2 V) studied here, it may not fully account for strong electron–electron correlations or incoherent processes that could become important at higher biases or in strongly localized systems. Moreover, excited-state transport channels and transitions between many-body states that may emerge at higher biases are beyond the scope of the present single-particle framework.

The values capture the essential bonding and antibonding interactions between metal (M), carbon (C), and termination (T) atoms. Also, semi-infinite electrodes are considered for better uniformity and stability of the device system, and with the semi-infinite electrode self-energies evaluated using the Sancho–Rubio recursive method to ensure numerical convergence. The retarded Green’s function of the device region is expressed as:^29^

1 $$\:{\mathbf{G}}^{\mathrm{r}}(\mathrm{E},{\mathrm{V}}_{\mathrm{b}\mathrm{i}\mathrm{a}\mathrm{s}}\text{})=\left[\right(\mathrm{E}+\mathrm{i}{\upeta\:})\mathbf{I}-{\mathbf{H}}_{\mathbf{D}}\text{}({\mathrm{V}}_{\mathrm{b}\mathrm{i}\mathrm{a}\mathrm{s}\mathrm{}})-{\boldsymbol{\Sigma\:}}_{\mathbf{L}}\mathrm{}-{\boldsymbol{\Sigma\:}}_{\mathbf{R}}\text{}{]}^{-1}$$

where **H**_**D**_ ​ is the device Hamiltonian under bias, η is a positive infinitesimal, and **Σ**_**L, R**_ ​ are the self-energies of the electrodes. From this, the transmission spectrum is obtained as:^30^

2 $$\:\mathrm{T}(\mathrm{E},{\mathrm{V}}_{\mathrm{b}\mathrm{i}\mathrm{a}\mathrm{s}}\text{})=\mathrm{T}\mathrm{r}\left[{\boldsymbol{\Gamma\:}}_{\mathrm{L}}\text{}{\mathbf{G}}^{\mathrm{r}}{\boldsymbol{\Gamma\:}}_{\mathrm{R}}\text{}{{\mathbf{G}}^{\mathrm{r}}}^{†}\right]$$

with ${\Gamma _\alpha }=i({\Sigma _\alpha } - \Sigma _{\alpha }^{\dag })$. The current is then calculated using the Landauer–Büttiker formula:^31^

3 $$\:\mathrm{I}\left({\mathrm{V}}_{\mathrm{b}\mathrm{i}\mathrm{a}\mathrm{s}}\text{}\right)=\frac{2\mathrm{e}}{\mathrm{h}}\text{}{\int\:}_{-{\infty\:}}^{+{\infty\:}}\mathrm{T}(\mathrm{E},{\mathrm{V}}_{\mathrm{b}\mathrm{i}\mathrm{a}\mathrm{s}}\text{})\left[\mathrm{f}\right(\mathrm{E}-{{\upmu\:}}_{\mathrm{L}}\text{})-\mathrm{f}(\mathrm{E}-{{\upmu\:}}_{\mathrm{R}}\text{}\left)\right]\mathrm{d}\mathrm{E}$$

where **f(E)** is the Fermi–Dirac distribution, and ${\mu _{L/R}}= \pm {{{V_{bias}}} \mathord{\left/ {\vphantom {{{V_{bias}}} 2}} \right. \kern-0pt} 2}.$.

### Inclusion of strain

To investigate strain effects, uniaxial strain was applied along the X-direction by modifying the in-plane lattice constant as:

4 $$\:{\mathrm{a}}_{\mathrm{x}{\prime\:}}\text{}={\mathrm{a}}_{\mathrm{x}}\text{}(1+{{\upepsilon\:}}_{\mathrm{x}}\text{})\text{}\text{},\text{}\text{}{\mathrm{a}}_{\mathrm{y}{\prime\:}}\text{}={\mathrm{a}}_{\mathrm{y}}\text{},\text{}\text{}{\mathrm{a}}_{\mathrm{z}{\prime\:}}\text{}={\mathrm{a}}_{\mathrm{z}}$$

where ε_x_​ is the applied strain (positive for tensile, negative for compressive). The corresponding interatomic distances were recalculated as:

5 $$\:{\mathrm{R}}_{\mathrm{i}\mathrm{j}{\prime\:}}\text{}=\sqrt{\left({\mathrm{x}}_{\mathrm{i}\mathrm{j}}\text{}\right(1+{{\upepsilon\:}}_{\mathrm{x}\text{}}){)}^{2}+{{\mathrm{y}}_{\mathrm{i}\mathrm{j}}}^{2}\text{}+{{\mathrm{z}}_{\mathrm{i}\mathrm{j}}}^{2}}\text{}$$

In this study, strain is applied uniaxially along a chosen Cartesian direction. The form shown in Eq. (5) is for strain along the x-axis (ε_x_). For uniaxial strain along y or z, the corresponding coordinate (y_ij_ or z_ij_) is scaled by its strain factor (1 + ε_y_ or 1 + ε_z_), while the other coordinates remain unchanged.

Since the tight-binding hopping parameters t_ij_ depend exponentially on bond length, the strained hopping’s were updated exponential Slater–Koster scaling:^32^

6 $$\:{{\mathrm{t}}^{{\prime\:}}}_{\mathrm{i}\mathrm{j}}\text{}={\mathrm{t}}_{\mathrm{i}\mathrm{j}\text{}}\mathrm{e}\mathrm{x}\mathrm{p}[-{\upbeta\:}\frac{({{\mathrm{R}}^{{\prime\:}}}_{\mathrm{i}\mathrm{j}}\text{}-{\mathrm{R}}_{\mathrm{i}\mathrm{j}}\text{})}{{\mathrm{R}}_{\mathrm{i}\mathrm{j}}}\text{}]$$

Because different bonds in MXenes involve electronic overlaps of different orbital character, we assigned β-values according to the physical nature of each interaction. For C–C bonds within the carbide layer, where the covalent overlap is comparable in stiffness to π-like bonding in graphene, we used β(C–C) = 3.3^33,34^. For metal–carbon bonds (Ti–C or Sc–C), which are dominated by directional d–p interactions and therefore exhibit stronger sensitivity to lattice deformation, we employed β(M–C) = 4.5^35–37^.

### Simulation parameters

The energy grid for transport integration was sampled within [− 2, 2] eV with a resolution of ΔE = 0.001 eV. We varied the bias voltage from 0 to 2 V, with small steps of 5 mV in the low bias regime (V < 0.2) and larger steps of 50 mV in biases above 0.2 V. Temperature effects were included by evaluating the Fermi–Dirac distribution at selected values of T = 4, 77, and 300 K.

This computational framework allows for the analysis of density of states (DOS), transfer spectra, and current-voltage (I-V) characteristics under combined bias, temperature, and strain conditions. Therefore, by examining all of the above, we expect to gain a proper physical view of the strain effects on nanoelectronics devices based on MXenes.

The uniaxial strain range considered in this study extends up to **± 6%**, which is often adopted in theoretical investigations to probe the limits of structural and electronic tunability in 2D materials. Within a linear elastic approximation, the corresponding stress can be roughly estimated using Hooke’s law, σ = *E* ⋅ ε, where E is the in-plane Young’s modulus. Reported values of E for Ti₃C₂T_x_ MXenes range from about 330GPa (experimental) to nearly 480GPa (theoretical predictions). This yields a stress level of approximately 20–29GPa for ε = 6%. These values approach or slightly exceed the experimentally measured fracture strength of Ti₃C₂T_x_ monolayers (~ 17GPa), suggesting that such a high strain would be close to the mechanical failure limit for a free-standing, defect-free sheet^6,19,38^.

In practical device architectures, however, MXene layers are typically integrated on compliant substrates (e.g., elastomers) or embedded in polymeric composites, which significantly redistribute the stress and allow larger apparent strains without fracture. In such configurations, strains of several percent are routinely applied in flexible strain sensors and stretchable electronics. Therefore, while ± 6% strain represents an upper theoretical bound for intrinsic material response, it remains technologically relevant^18,19^.

## Results and discussion

**Fig. 2:**
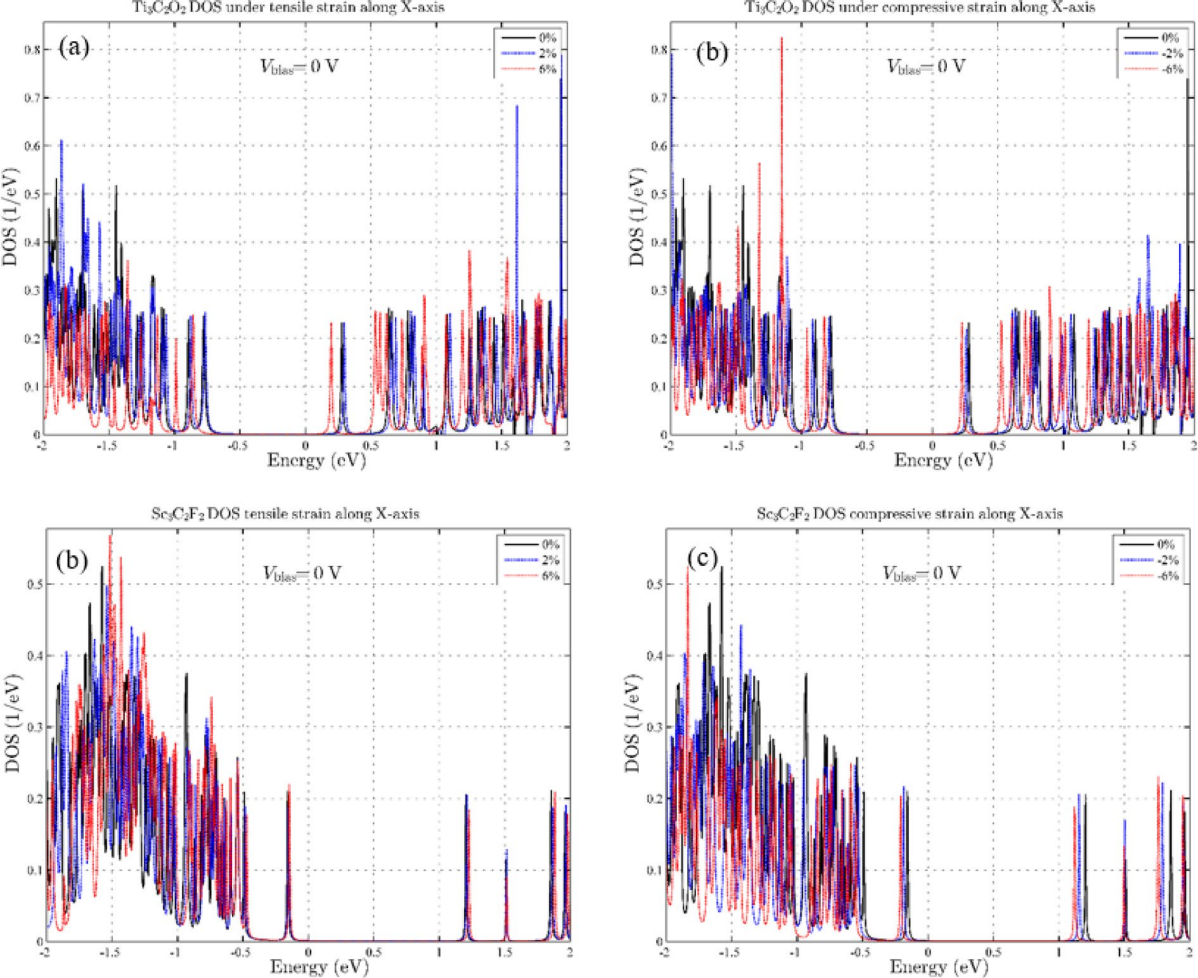
Density of states for strain along the x-axis: (**a**) Tensile strain on Ti₃C₂O₂, (**b**) Compressive strain on Ti₃C₂O₂, (**c**) Tensile strain on Sc₃C₂F₂, (**d**) Compressive strain on Sc₃C₂F₂.

**Fig. 3:**
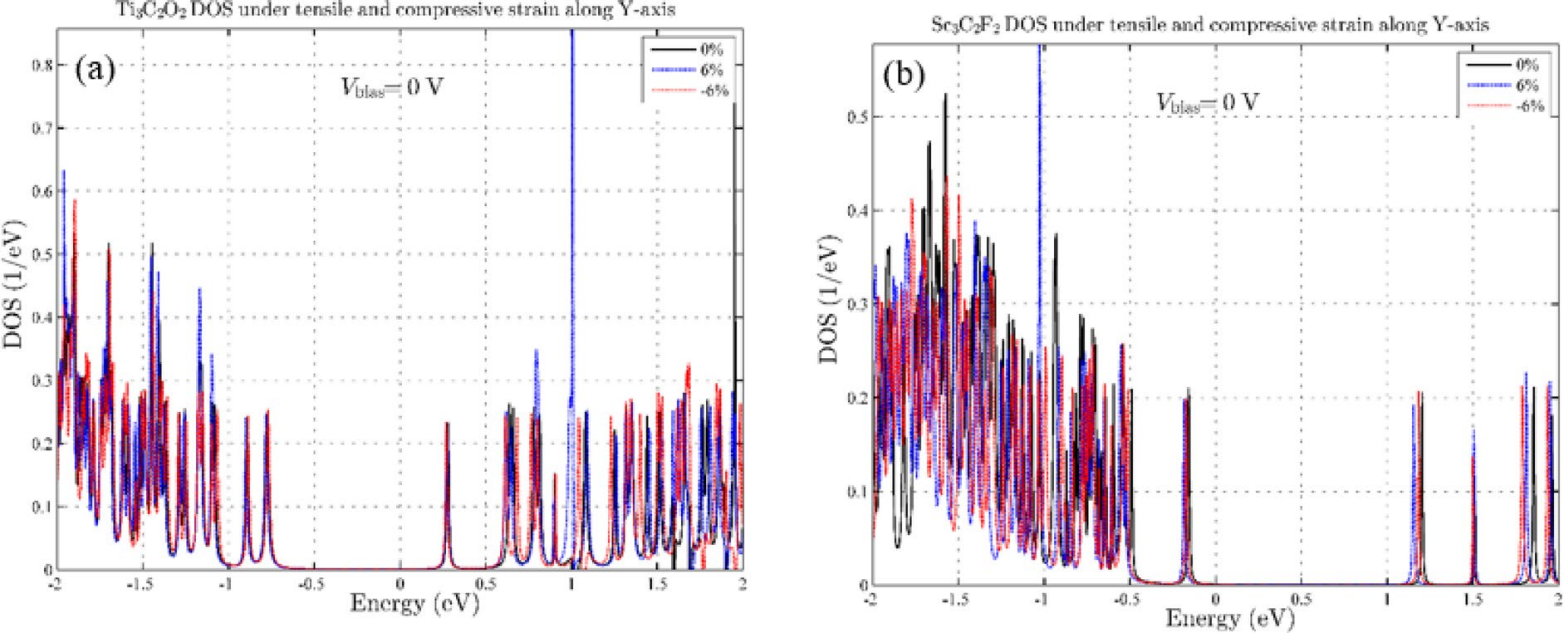
Density of states diagram for strain effect in the y-axis. (**a**) for Ti_3_C_2_O_2_ and (**b**) for Sc_3_C_2_F_2_.

In the initial stage, we investigate the effects of tensile and compressive strain in all three directions of the coordinate axes. According to Fig. 2, parts (a) and (b), for Ti_3_C_2_O_2_, the tensile and compressive effects in the x-axis, it causes a shift in energy levels and, as a result, a decrease in the band gap near the Fermi level, which is highest at a stretch of 6%. On the other hand, the effect of compressive strain does not significantly change the band gap and only causes a shift in the energy levels. For Sc_3_C_2_F_2_, as for Ti_3_C_2_O_2_, strain effects in the x-axis have caused energy level shifts and still, the largest energy level shift is due to strain, which is ± 6%. But in general, we can be said, unlike Ti_3_C_2_O_2_, in Sc_3_C_2_F_2_, the effects of tensile strain are less than the effects of compressive strain in the x-axis (Fig. 2c and d). To understand the effects of strain in-plane, we also examine the strain in the y-axis. As shown in Fig. 3, the effects of tensile and compressive strain in the y-axis are negligible and a small energy shift has occurred in the DOS. This indicates that the effects of tensile and compressive strain in the y-axis have practically no effect either near or far from the Fermi level, and the Sc_3_C_2_F_2_, and Ti_3_C_2_O_2_ MXene device is insensitive to this strain.

**Fig. 4:**
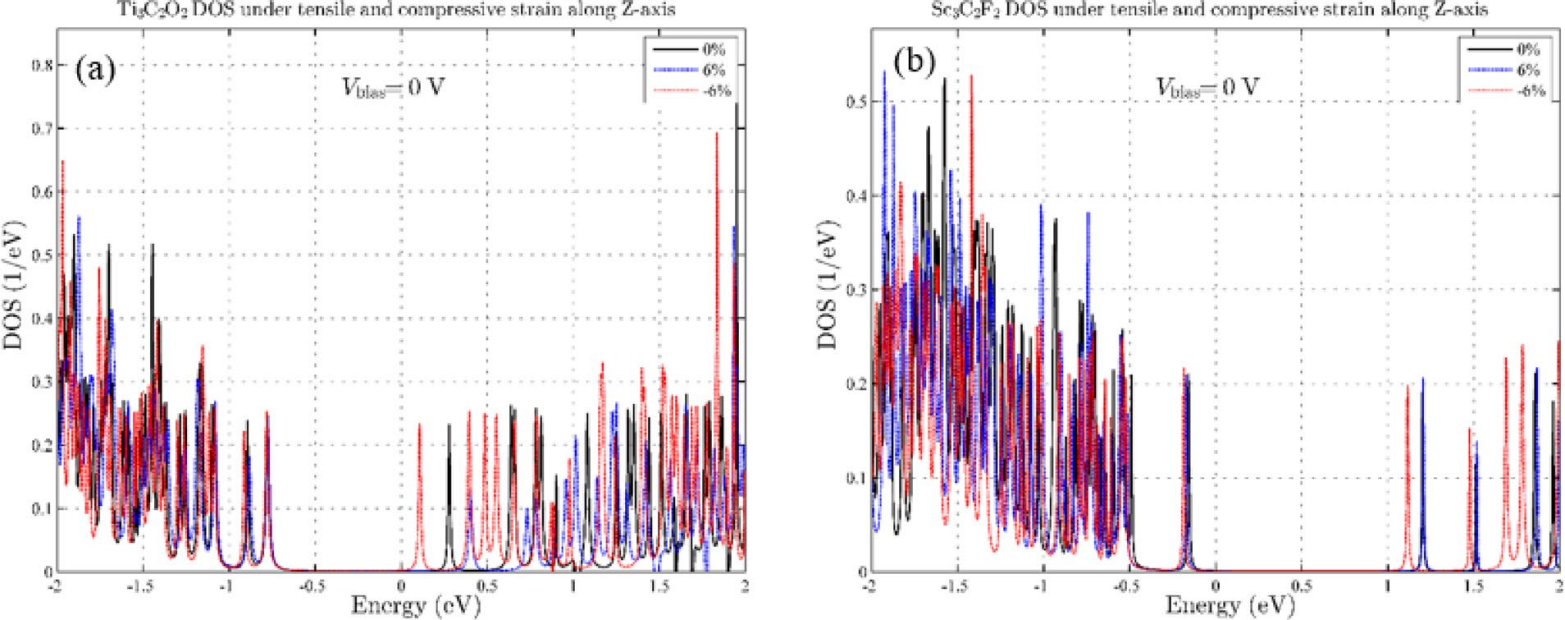
Density of states diagram for compressive strain effect in the Z-direction. (**a**) for Ti_3_C_2_O_2_ and (**b**) for Sc_3_C_2_F_2_.

**Fig. 5:**
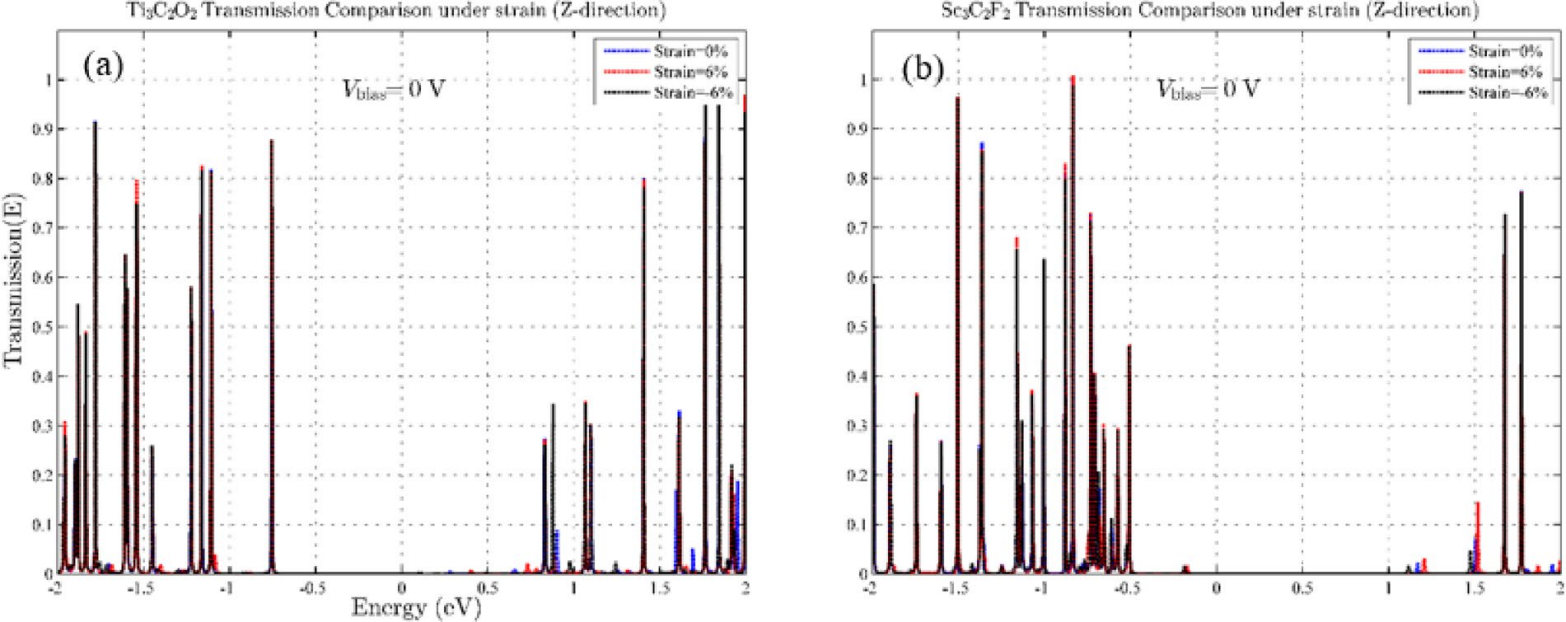
Transmission spectra for − 6% compressive and + 6% tensile strain effect in the Z-direction. (**a**) for Ti_3_C_2_O_2_ and (**b**) for Sc_3_C_2_F_2_.

Since in structures with small dimensions, the effects of strain perpendicular to the device plane are usually considered more important, we also investigate the effect of tensile and compressive strain in the z-direction^39,40^. Figure 4 shows the effects of a compressive strain of -6% and a tensile strain of + 6% applied to the two MXene device structures. It is clearly evident that strain effects in the z-axis have more pronounced effects on changes in the density of states. Our results for Ti_3_C_2_O_2_ show more pronounced changes due to strain in the z-axis than for Sc_3_C_2_F_2_. In Ti_3_C_2_O_2_, we see more changes with increasing pressure, and the number of available states near the Fermi level increases (Fig. 4a). To gain deeper insight into the effects of strain on electron transport, and to determine whether variations in the available states influence this transport, we examine electron transmission. In Ti₃C₂O₂, the band gap increases with the application of compressive strain, and we see a p-type semiconductor channel with a lower gap. Since these results were obtained at zero bias voltage, we expect to see increased conductivity at this strain as the voltage increases. In contrast, in Sc_3_C_2_F_2_, we have the exact opposite result. The band gap of the channel decreases with tensile strain, and we see an n-type semiconductor channel with a reduced energy gap. Next, in order to investigate the effect of strain on transmission, we investigate the effect of strains that had the greatest impact on the density of states by drawing and analyzing the T(E) spectra. Figure 5 presents the transmission spectra of Ti₃C₂O₂ and Sc₃C₂F₂ structures subjected to 6% compressive and tensile strain applied perpendicular to the plane. According to Fig. 5, although the DOS shows noticeable changes under strain, the transmission comparison shows that the conductivity, without applying bias voltage, remains almost stable near the Fermi level.

**Fig. 6:**
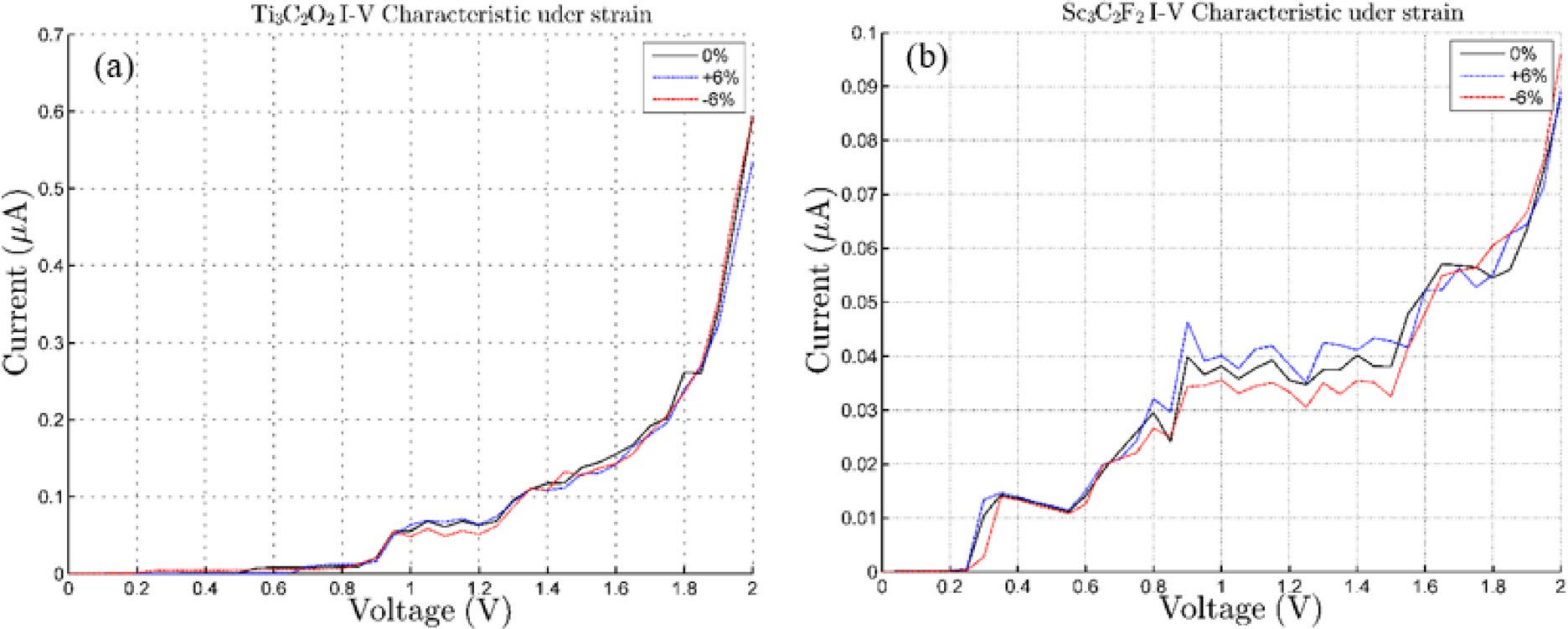
I-V characteristic for compressive and tensile strain effect in the Z-direction. (**a**) for Ti_3_C_2_O_2_ and (**b**) for Sc_3_C_2_F_2_. +6% is shown in blue, -6% in red, and the strain-free structure in black.

Although small transmission peaks appear near the Fermi level, indicating the shift of energy levels near the Fermi level, the device channel is still semiconducting. In order to evaluate the impact of mechanical deformation on electronic transport, we calculated the current–voltage (I–V) characteristics of Ti₃C₂O₂ and Sc₃C₂F₂ under uniaxial strain. Figure 6 show the resulting I–V curves for tensile and compressive strain values up to ± 6% along the Z-directions. For Ti₃C₂O₂, exactly as expected from the density of states diagram, by applying compressive strain, the band gap decreases, the available states in the conduction band approach the Fermi level, and the current starts to increase from a lower voltage. By applying tensile strain, the band gap increases and as a result the conduction band is doubled from the Fermi level, which causes the electric current to start at a higher voltage. By increasing the voltage from 1.6 to 2 V, due to the sudden increase in current, we observe a current due to quantum tunneling, where the increase in current with the application of compressive strain is greater than that with tensile strain.

The maximum relative change in the current does not exceed about 10% in the studied bias range. This result indicates that the charge transport channels in Ti₃C₂O₂ are robust and largely unaffected by changes in the lattice structure.

In contrast, Sc₃C₂F₂ shows an even more stable I–V behavior under the same strain range. The electric current in the state of tensile and compressive strain and without strain begins to increase at approximately the same voltage. The electric current in the tensile and compressive strain and without strain states starts to increase from approximately the same voltage and in all three I-V characteristics we see negative differential resistance.

These findings highlight a clear material-dependent response: while both MXenes maintain overall robust conduction under moderate strain, Ti₃C₂O₂ exhibits slightly higher sensitivity than Sc₃C₂F₂ and Under strain, the device turns on at a lower voltage.

## Concluding remarks

In view of the obtained results, we investigated the effects of tensile and compressive strain on the structure of Ti_3_C_2_O_2_ and Sc_3_C_2_F_2_ MXene nanodevices. Our results showed that applying strain parallel to the device channel has limited effects on its electronic behavior. In contrast, the effects of strain perpendicular to the channel of the Ti_3_C_2_O_2_ device cause a decrease in the energy gap, resulting in sensitivity to compressive strain at lower voltages. Hence, we suggest that this material can be used in pressure-sensitive devices. The slight changes observed near the Fermi surface in Sc_3_C_2_F_2_ indicate that strain does not significantly alter the conduction channels of these MXene. From a practical perspective, this resistance to deformation makes Sc₃C₂F₂ promising candidate for applications in flexible and strain-resistant nanoelectronics.

## Data Availability

The datasets used and/or analysed during the current study are available from the corresponding author on reasonable request.
